# Development, Optimization and Evaluation of a Sensitive Enzyme-Linked Immunosorbent Assay (ELISA) Prototype for Detection of Chicken-Based IgY Polyclonal Antibodies against Toxins of *D. polylepis* Venom

**DOI:** 10.3390/antib13030050

**Published:** 2024-06-21

**Authors:** Stephen Wilson Kpordze, Gideon Mutie Kikuvi, James Hungo Kimotho, Victor Atunga Mobegi

**Affiliations:** 1 Department of Molecular Biology and Biotechnology, Pan African University Institute for Basic Sciences, Technology and Innovation (PAUSTI), JKUAT-Juja Campus, Nairobi 62000-00200, Kenya; 2 Spanish Laboratory Complex, University for Development Studies, Nyankpala Campus, Tamale TL 1350, Ghana; 3 Department of Environmental Health and Disease Control, Jomo Kenyatta University of Agriculture and Technology, JKUAT-Juja Campus, Nairobi 62000-00200, Kenya; kikuvi@itromid.jkuat.ac.ke; 4 Kenya Medical Research Institute, Off Raila Odinga Way, Nairobi 54840-00200, Kenya; jhkimotho@kemri.org; 5 Department of Biochemistry, University of Nairobi, Chiromo Campus, Nairobi 30197-00100, Kenya; vatunga@uonbi.ac.ke

**Keywords:** chicken-based IgY, ELISA, optimization, *D. polylepis* venom, antivenoms

## Abstract

Life-threatening medical issues can result from snakebite, and hence this is a public health concern. In many tropical and subtropical nations such as Kenya, where a wide variety of poisonous snakes are prevalent, diagnosis of snakebite in health facilities is imperative. Different antivenoms are needed to treat the venom of different snake species. Nonetheless, it might be difficult for medical professionals to identify the exact snake species that envenomated a patient due to the similarities of several snake envenomations’ clinical symptoms. Therefore, the necessity for an assay or technique for identifying venomous species is critical. The current study sought to develop a sensitive ELISA prototype for the detection of *D. polylepis* venom in Kenya using generated chicken-based IgY polyclonal antibodies. Serum samples containing specific chicken-based IgY antibodies previously raised against *D. polylepis* venom toxins were used in the assay development. ELISA parameters were optimized, and the developed assay was assessed for applicability. The limit of detection (LoD) of the ELISA for neurotoxic venoms was determined to be 0.01 µg/mL. Successful discrimination between neurotoxic and cytotoxic venoms was achieved by the ensuing inhibition ELISA assay. The developed assay showed the capability of identifying venoms in blood samples (from spiked and venom-challenged blood samples) of BALB/c mice, providing compelling evidence of the strategy’s usefulness. This assay could help physicians diagnose and manage victims of snakebites through the evaluation of clinical samples.

## 1. Introduction

In the tropical agrarian world, snakebite represents a significant medical and public health concern, with an estimated worldwide burden of 1.8 to 2.7 million yearly, with more than 138,000 fatalities and more than 400,000 victims with permanent disabilities annually [1,2]. Yearly, 314,000 cases of snakebite envenomations resulting in 5900–14,600 amputations and 7000–32,000 fatalities in sub-Saharan Africa are recorded [1]. Given its impact on world health, snakebite is recognized by the World Health Organization as a category A neglected tropical disease [3].

In tropical regions where human–snake encounters are more likely due to a confluence of environmental, geographic and demographic variables, snakebite is a serious public health concern [3]. Even with the recent resurgence of interest in snakebite worldwide, the actual burden is still unknown. Snakebite may not have been prioritized as a leading cause of death and disability sooner due to inadequate reporting and data collection, excessive dependence on data from health facilities, and the creation of modeled estimates from regional statistics [3,4]. In addition, reports from studies indicate that up to 70% of snakebite incidents go unreported [5,6].

Kenya is one of the sub-Saharan African countries battling with high incidence of snakebites. A facility-based research study reported that the annual incidence of snakebite in Kenya ranges from 1.9 to 67.9 cases per 100,000 people, with Samburu county having one of the highest rates at 66 cases per 100,000 inhabitants (with 1406 snakebites and 88 deaths from snakebites per year) [7]. Also, a different community-based survey revealed a significantly higher yearly incidence of snakebite in Kilifi County with 150 per 100,000 inhabitants [8]. A recent study on the prevalence and mortality rate of snakebite found 81 snakebite victims and 5 snakebite-related deaths in different geographical regions of Kenya. Within the past 5 years, there was a 2.2% (95% CI 1.4–3.4) prevalence of snakebite and 138 (95% CI 44–322) per 100,000 people died [4]. A total of 7772 Kenyans were bitten by snakes between the years of 2007 and 2016, of which 614 died [9]. It is estimated by official statistics that between 15 and 20 Kenyans are bitten by snakes daily; some of these injuries end in amputation of a limb or even death [10]. From the ‘guidelines for the prevention diagnosis and management of snakebite envenoming’ in Kenya by the Ministry of Health, there are 140 known snake species in Kenya, out of which 29 are venomous. A total of these 29 species are of medical importance due to the cause of death in extreme cases or injuries, and 9 of these cause bites that need medical attention [11]. Among these species is the *D. polylepis*, which is a highly dangerous venomous snake behind major recorded fatalities in Kenya [11]. Also, *B. arietans* and *N. ashei* constitute medically important snake species in sub-Saharan African countries such as Uganda, Tanzania, Somalia and Kenya, and are therefore implicated in snakebites with associated mortality, disability and morbidity [12,13]. Hence, they have been employed in this study for comparative testing.

Antivenom is a key treatment option for snakebite, but lack of access and availability in Africa with Kenya not an exception remains a significant public health challenge, and even very costly when available [14,15]. The wholesale price of antivenom vials ranges from USD 18 to USD 200 in sub-Saharan Africa, and an effective antivenom treatment regimen typically costs USD 124 on average [16]. Amid these challenges are limited or ineffective diagnostic approaches for snakebite detections. It is, therefore, crucial to detect serum antibodies or venom toxins using a sensitive and specific diagnostic assay to enable appropriate treatment.

Most clinicians or researchers from different parts of the world have carried out snake venom detection in serum samples taken from snakebite victims for medical and scientific reasons [17,18,19,20,21,22,23]. However, the inability to accurately identify the offending snake and diagnose envenoming are two major clinical issues in the management or treatment of snakebite envenomations. Detection assay kits, which could have helped in most Kenyan hospitals remain a challenge. Similar to other snake-endemic countries, many victims of snakebites do not visit medical centers with the snake that bit them [24]. In cases where a snake is presented, similarities in the morphology of the specimen hamper identification [25,26]. On the other hand, the methods of identifying the snake that are commonly employed, while not always reliable, include using the descriptions given by victims or their families/companions, or recognizing it through a preserved specimen and photo examination [24,27,28]. Syndromic identification of the offending snake is an additional strategy; however, the specificity and sensitivity of this method are limited [24,29]. Both toxinology research and clinical therapy are hampered by the inability to identify the snake, and erroneous identification of the snake causing the problem may result in improper patient management, through both over- and under-treating patients, which may cause negative effects [24,30]. More so, the inability to identify the causative snake species taints clinical investigations ranging from case reports to randomized controlled trials [31].

In Kenya, few preclinical and no clinical investigations have used enzyme immunoassays to detect the venom of Kenyan medically important snakes, and its employment in regular clinical practice in this regard is lacking [13]. Enzyme immunoassay is the most widely used technique for detecting venom in biological materials because of its excellent analytical sensitivity. The application of enzyme-linked immunosorbent assay (ELISA) to snake venom studies has also increased venom immunotherapy’s safety and efficiency. This technique has been utilized more frequently since its introduction in 1977 [19] in several nations for routine diagnostic procedures, as well as clinical toxicology research [32,33,34]. In this study, we aim to develop a sensitive enzyme-linked immunosorbent assay (ELISA) to detect and quantify Kenyan Black mamba (*D. polylepis*) venom using IgY antibodies raised in chicken serum samples. In order to determine the specificity of the assay, this study also sought to determine how well the assay performs in terms of cross-reactivity with different snake venoms, spiked blood samples and venom-challenged mice.

## 2. Materials and Methods

### 2.1. Reagents

Rabbit anti-chicken IgG/HRP conjugated antibody, 3,3′,5,5′-tetramethylbenzidine (TMB) and bovine serum albumin (BSA) (Solarbio, Beijing, China), o-phenylenediamine dihydrochloride (OPD) 5 mg-tablet, Tween-20, Freund’s incomplete adjuvant (FIA) and Freund’s complete adjuvant (FCA) (Sigma-Aldrich, St. Louis, MI, USA), OPD powder (ThermoFishers, Norristown, PA, USA), citric acid monohydrate ((HOC(COOH)(CH_2_COOH)_2_·H_2_O, mw = 210.14), di-sodium hydrogen orthophosphate 12-hydrate (Na_2_HPO_4_·12H_2_O, mw = 358.14), hydrogen peroxide (H_2_O_2_) and sulfuric acid were obtained from the manufacturers. The other chemicals or reagents used were analytical grade and bought from local companies.

### 2.2. Animals

Seventeen-week-old Isa-Brown layer hens (weighing 1.7–1.8 kg each, *n* = 5), and in good health were purchased from a local poultry farm, and used for raising IgY in serum against *D. polylepis* venom. The hens were kept at the Small Animal Facility for Research and Innovation (SAFARI) in Jomo Kenyatta University of Agriculture and Technology (JKUAT) in their individual cages with the provision of standard water and food. Both female and male BALB/c mice weighing between 28 and 30 g each and, at eight weeks old, were obtained from the Kenya Institute of Primate Research (IPR) and used for the venom-challenged assay, as well as sampling blood for spike samples during ELISA assays. Purchased mice were maintained at the animal house in the Kenya Medical Research Institute with the provision of food and water *ad libitum.*

### 2.3. Venoms and Antivenoms

The various venoms used in this study were from *D. polylepis*, 2-homologous *Dendroaspis* sp. (*D. angusticeps* and *D. jamesoni*) and 2-heterologous *Dendroaspis* sp. (*B. arietans* and *N. ashei*). The *D. polylepis* venom was used in raising the IgY antibodies in chicken and for the ELISA assays. Other homologous and heterologous snake venoms were used for ELISA specificity and toxins detection assays. Venoms were obtained through donations from the Kenya Medical Research Institute (KEMRI) and archives of Prof. Joseph K. Gikunju at the Department of Medical Laboratory Science, JKUAT, Nairobi, Kenya. Crude venoms were lyophilized and stored at −20 °C until use. Two antivenoms purchased commercially for this study included VINS™ (African IHS, B. NO: 07AS21006; expiry date: 07/2025) and PANAF-Premium™ (Pan Africa, B. NO: PANAF-027; expiry date: 08/2027), and were reconstituted following the manufacturer’s instructions.

### 2.4. Schedule of Immunizations for Hens

For the initial immunization, each of the 4 hens received an intramuscular injection of 0.5 mL of saline containing 0.341 mg of *D. polylepis* venom emulsified with an equal volume of Freund’s complete adjuvant (FCA) at different sites in their breast region.

Subsequent booster doses consisted of 0.5 mL of saline containing 0.54 mg, 1.08 mg, 2.16 mg and 2.16 mg of *D. polylepis* venom, emulsified with an equal volume of Freund’s incomplete adjuvant (FIA), administered on days 14, 35, 56 and 77, respectively. The control group’s hen was immunized intramuscularly with only 0.5 mL saline at multiple sites of the breast region. Weekly blood samples collected before and after first immunization, were processed for serum and stored at −20 °C for further use. Also, eggs were collected for IgY antibody extraction and confirmation.

### 2.5. ELISA Parameters Optimization

An indirect ELISA approach, as reported by Islam & Jones [35] and Manson et al. [13] with some modifications was used to optimize for the rabbit anti-chicken IgG/HRP-conjugated antibody, antigen coating concentration and substrate sensitivity.

#### 2.5.1. Optimizing for Secondary Antibody

Following the methods for ELISA from reports as indicated earlier in Islam & Jones [35] and Manson et al. [13], *D. polylepis* (Black mamba) venom was diluted in coating buffer (0.1 M carbonate–bicarbonate, pH 9.6) to obtain 1 µg/mL concentration. From this preparation, 100 µL was added to each well of a 96-well plate (Maxisorp plate, NUNC, Roskilde, Denmark), covered and incubated overnight at 4 °C. The antigen-coating buffer was removed, washed 3 times with 3 min in between each wash using 200 µL of washing buffer (PBS, containing 0.05% Tween-20, pH 7.4) and excess water was blotted on a tissue paper. Each well of the washed plate was then blocked with 200 µL of blocking buffer (2% BSA + washing buffer), incubated at room temperature for 1 h and washed as previously described. Pooled serum (containing primary antibody) sample was diluted at 1:1000 in dilution buffer (PBS, containing 0.5% BSA, pH 7.4), and 100 µL of diluted samples added to wells in duplicates before incubating the plate at 37 °C for 1 h, sample removed and washed. A 2-fold serial dilution was performed for the rabbit anti-chicken IgG/HRP secondary antibody (from 1:2000 to 1:256,000), and 100 µL of diluted antibody was added to wells, incubated for 1 h at 37 °C, removed and washed 3 times. A 200 µL of TMB substrate solution was added to each well and incubated at room temperature in the dark for 15–12 min, and the reaction stopped upon adding 50 µL of 2 M H_2_SO_4_ solution. Absorbance was measured at 450 nm. However, preliminary secondary antibody dilutions were tried before settling on the range indicated.

#### 2.5.2. Antigen Coating Optimization

Based on the 1–10 µg/mL antigen (*D. polylepis* venom) concentration recommended range [13,35], using a checkerboard approach, different antigen concentrations were tested in parallel. The 1, 2, 4, 6, 8 and 10 µg/mL concentrations of *D. polylepis* venom in coating buffer were used to coat plates and tested while evaluating two secondary antibody dilution (1:5000 and 1:10,000) combinations towards achieving optimal antigen concentration–detection antibody dilution combinations. Serum sample dilutions, blocking and washing of plate, substrate addition and absorbance reading were all performed as previously described (see Section 2.5.1).

#### 2.5.3. ELISA Substrates Optimizations

The previously described indirect ELISA method was employed to compare tetramethylbenzidine (TMB) and o-phenylenediamine dihydrochloride (OPD) substrates performance. The antigen coating (1 µg/mL *D. polylepis* venom in coating buffer), pooled serum sample (containing the primary antibody, dilution 1:1000), and rabbit anti-chicken IgG/HRP secondary antibody (dilution 1:5000) blocking and washing were carried out as previous. A 100 µL of TMB substrate solution was added to each well and incubated in the dark at room temperature for 15–20 min, whereas 200 µL of OPD substrate solution (containing 0.1 M citric acid monohydrate, plus 0.2 M di-sodium hydrogen orthophosphate 12-hydrate, pH 5.0; 5 mg OPD tablet, 30% hydrogen peroxide) was added to wells of OPD plate followed by 20–30 min incubation in the dark at room temperature. After the reaction was stopped, absorbance was read at 450 nm and 492 nm, respectively, for TMB and OPD plates.

#### 2.5.4. Primary Antibody (Serum) Dilution Optimization

All other procedures for the ELISA were the same as described previously, except for the coating plate with 1 µg/mL of antigen, the pooled serum sample dilutions (1:500, 1:1000, 1:2000 and 1:4000), the secondary antibody diluted at 1:5000 and the use of the optimized substrate (OPD).

### 2.6. ELISA Specificity and Sensitivity (Cut-Off Point) Determination

To evaluate the ELISA’s ability to distinguish between closely related and unrelated venoms, procedures from [35,36] were modified and employed. Briefly, two heterologous (*B. arietans* and *N. ashei*) and two homologous (*D. angusticeps* and *D. jamesoni*) venom samples were used to measure the assay’s specificity using the observed percent inhibition at different sample antigen concentrations (6.6–0.003 µg/mL). A 3-fold serial dilutions of sample antigens were performed in coating buffer for various venom concentrations, and 100 µL of the aliquot was used to coat the 96-well plate. The ‘No antigen control (NAC)’ row was included for each snake species venom. Blocking, using serum sample diluted at 1:2000, rabbit anti-chicken IgG diluted at 1:8000 and OPD substrate solution was performed using the previously described procedure. Percent inhibition was then calculated across all inhibitor concentrations for venoms, and patterns were compared between homologous and heterologous venom samples.

Additionally, sensitivity determination for the ELISA was assayed by coating the plate with crude *D. polylepis* venom with an initial concentration of 44 µg/mL to a final of 0.02 µg/mL. A 3-fold serial dilution was made, and samples were analyzed in duplicates. During this, 16 pre-immuned serum samples and sample buffer were analyzed as controls (negative controls, devoid of an analyte of interest), and were used in determining the limit of detection (LoD). Consequently, the assay’s limit of detection (LoD) was ascertained utilizing the formula below, which is based on the signal–noise approach [35]:

*LoD* = *concentration of antigen in well with* % *inhibition* > *mean* + 2 × *Standard Deviation of negative controls*

### 2.7. Inhibition ELISA for Detecting Toxins in Crude D. polylepis Venom

A modified procedure from Sharma et al. [36] was employed for this assay. Briefly:

Step 1: A 96-well plate (1) was coated with 100 µL of 1 µg/mL *D. polylepis* venom constituted in coating buffer and incubated overnight at 4 °C. On the following day, the solution was removed, the plate was washed 3 times, as previously described, and each well was blocked using 200 µL of blocking buffer. The blocked plate was incubated at room temperature for 1 h, washed as previously, and extra water was blotted out using tissue paper.

Step 2: A 44 µg/mL final concentration of sample antigen (*D. polylepis* venom) was prepared in blocking buffer, and 150 µL of the aliquot was added to wells of row A of a new uncoated plate (2). A volume of 100 µL blocking buffer was added to each well from row B up to H. Next, a 3-fold serial dilution was carried out; thus, 50 µL was transferred from row A to row B, until row G, with the extra 50 µL discarded. At a dilution of 1:2000, the serum sample (containing the primary antibody) was prepared in dilution buffer, and 100 µL each was added to wells including row H of the same plate (2). This was performed in duplicates. The plate (2) was incubated for 1 h at 37 °C.

Step 3: After that, the sample antigen and primary antibody-containing contents of plate (2) were transferred to plate (1), which had been coated, and incubated for 1 h at 37 °C. Again, the plate was washed 3 times, and 100 µL of diluted rabbit anti-chicken IgY/HRP in block buffer (1:8000) was added to each well, incubated for 1 h at 37 °C, and the washing step was repeated. After washing, 200 µL of OPD substrate solution was added to each well and incubated at room temperature in the dark for 20–30 min, and the reaction stopped upon adding 50 µL of 2 M H_2_SO_4_ solution. The plate was read using a plate reader (Multiscan EX reader, Thermo Scientific, Waltham, MA, USA) at 492 nm absorbance. Row H, which had no sample antigen but contained block buffer and primary antibody, was considered ‘No antigen control (NAC)’. Against the NAC absorbance, percentage inhibition for each well that contained a different concentration of the sample antigen was calculated as:

(1) $$Percent\;Inhibition=\frac{\left(NAC\;OD-Test\;Sample\right)}{NAC\;OD}\times 100$$

### 2.8. Evaluating Inhibition ELISA’s Ability for Toxin Identification in Other Dendroaspis spp. and Non-Dendroaspis spp. Venom Analysis

Crude venoms from two different *Dendroaspis* spp. (*D. angusticeps* and *D. jamesoni*) and two non-*Dendroaspis* spp. (*B. arietans* and *N. ashei*) were used to assess the inhibitory ELISA’s capacity to distinguish between venoms containing toxins of *Dendroaspis* spp. and those without. The above procedure (Section 2.7) was employed for this assay. However, sample antigen concentrations were prepared through serial dilution from 6.6 µg/mL to a final of 0.003 µg/mL. Incubation, washing, blocking, the addition of serum samples and a secondary antibody, and OPD substrate buffer solution were all carried out as described previously. The reaction was stopped by adding 50 µL of 2 M H_2_SO_4_ solution, the absorbance read was at 492 nm, and determining of percentage inhibition per well was performed.

### 2.9. Toxins Detection in D. polylepis Venom-Injected Mice

In this assay, the inhibition procedure described previously was followed (Section 2.7). Mice injected with crude *D. polylepis* venom were used to investigate the suitability of the inhibition ELISA in snakebite envenoming diagnosis. Five mice (BALB/c) were injected with *D. polylepis* venom (concentration of 0.1 mg/mL of venom in PBS). Blood samples were collected before injection, and used as negative control. After the venom challenge, mice were bled from the tail vein at 0.5, 1, 2, 4, 6, 8 and 24 h intervals. Samples were collected on ice, stored at 4 °C and immediately used after the last sample collection. Using the optimized parameters, the inhibition ELISA method was used as previously and percentage inhibition was calculated based on values of the optical density (OD) measured.

### 2.10. Toxins of D. polylepis Venom Detection in Spiked Samples

The inhibition ELISA procedure described previously was implemented to test the ELISA’s ability to detect toxins in blood samples that are spiked with *D. polylepis* venom. Despite similar conditions, the steps and components of the assay as previously carried out, and the sample collection and processing were different. Briefly, initial blood samples were collected from five BALB/c mice and used as a negative control or ‘No antigen control (NAC)’. To spike the samples, blood samples collected from mice tail veins were sustained at 0.5, 1, 2, 4, 6, 8 and 24 h intervals. An equivalent volume of reconstituted crude *D. polylepis* venom (concentration of 1 mg/mL) was added to blood samples right after they were collected. Collected samples were stored and assayed as previously indicated. Considering both the individual OD values and the NAC, the percentage inhibition was calculated.

### 2.11. Detection of Commercial D. polylepis Snake Antivenom Using Developed ELISA

We used a modified indirect ELISA assay method by Liu et al. [33] to assess its capacity to identify antibodies produced against toxins of *D. polylepis* and other snake venoms in two commercial antivenoms: PANAF-Premium^TM^ (Pan Africa, Premium Serums and Vaccines PVT Ltd, Maharashtra, India) and VINS^TM^ (African HIS, Vins Bioproduct Ltd, Telangana, India) polyvalent antivenoms. Briefly, a 96-well plate was coated using 100 µL of a 1 µg/mL antigen of *D. polylepis* venom in coating buffer, left overnight at 4 °C incubation. Plate wells were emptied after incubation, washed and blocked with 200 µL blocking buffer following 1 h incubation at room temperature, as previously. The block plate was emptied and washed 3 times at 3 min intervals. For each antivenom, a 9-point of 2-fold serial dilutions (from 1:1000 to 1:256,000) were performed in dilution buffer, 100 µL of each aliquot was added to the respective well in duplicates, and they were finally incubated for 1 h at 37 °C. The washing step was repeated after incubation and 100 µL of diluted (1:8000) rabbit anti-chicken IgG/HRP in blocking buffer was added to wells. The plate with secondary antibody suspension was incubated at 37 °C for 1 h, emptied and washed as previously. After this, 200 µL of prepared OPD substrate solution was added to each well, covered with an adhesive seal and incubated in the dark at room temperature for 20–30 min. The reaction was stopped after incubation using 50 µL of 2 M H_2_SO_4_. The plate was read at an absorbance of 492 nm.

### 2.12. Data Management

Data obtained from assays were entered into Microsoft Excel 2019 and analyzed using OriginPro version 2024 “https://www.originlab.com/myOriginDownload” (accessed on 1 February 2024). Substrate sensitivity analysis was carried out using a paired sample t-test. Group mean differences from the detection of other snake venoms, venom detection in spiked blood and blood of venom-challenged mice were ascertained using one-way ANOVA. Tukey’s multiple comparison test was used to compare the means within the comparisons, and statistical significance was determined (where *p*-value < 0.05).

## 3. Results

### 3.1. Optimization Assays of ELISA Parameters

**Figure 1:**
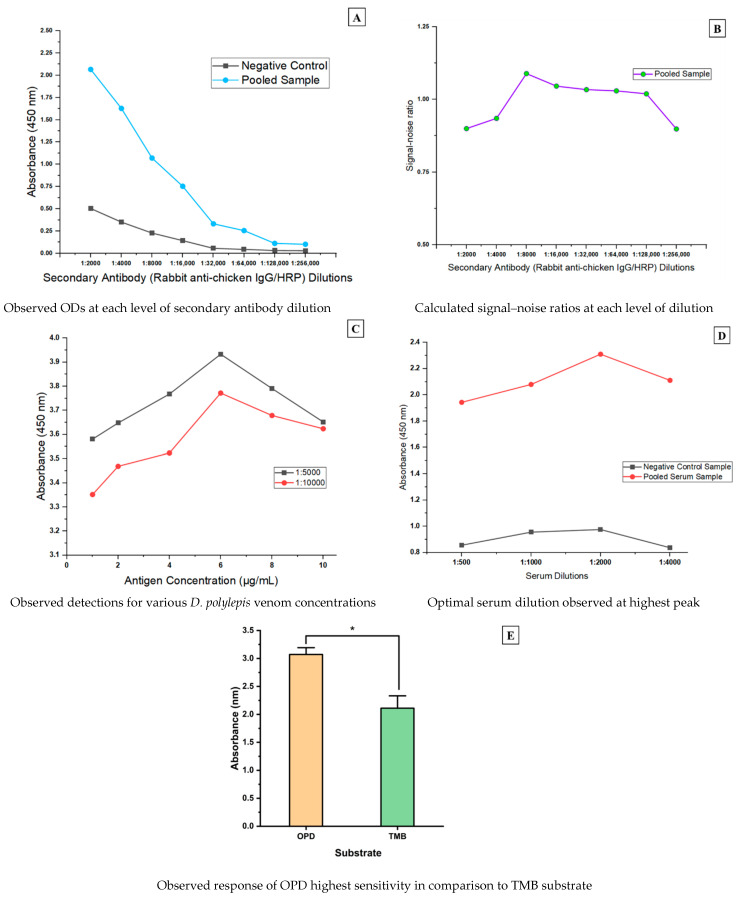
Determination of optimal rabbit anti-chicken IgG/HRP conjugate dilution, antigen coating, substrate and serum (pooled sample) dilution. (**A**) = trend of OD responses for secondary antibody dilutions; (**B**) = computed signal noise for secondary antibody response at each dilution; (**C**) = diluted antigen concentration responses; (**D**) = assessing optimal serum dilution; (**E**) = comparison of substrates sensitivity. * indicates a statistical difference.

An eight-point two-fold serial dilution was performed to find the optimal dilution of rabbit anti-chicken IgG/HRP conjugated antibody for the assay and was guided by the manufacturer’s recommended dilution of 1:10,000. Pooled serum samples from *D. polylepis* venom-immunized and PBS-immunized layer hens were used as positive and negative controls. From the ELISA parameters optimization assays, the immune response was detected at an OD of 2.067 value for venom-immunized chicken and an OD value of 0.505 for the negative control sample at the lowest rabbit anti-chicken IgG/HRP dilution of 1:2000. It was revealed that the detection of response as in the case of target antibody-containing serum sample was dilution-dependent. The negative control sample showed very little variation in detection and had saturation starting at the 1:32,000 dilution (Figure 1A). However, the highest signal–noise ratio for the secondary antibody conjugate was obtained at 1:8000 dilution and was determined by computing ratios of the absorbance readings of sample-negative control for each dilution (Figure 1B).

From the recommendation by Islam and Jones [35], the established antigen coating concentrations for this study showed increasing detection with a peak at 6 µg/mL and decreased with increasing concentration. However, at a coating of low antigen concentration of 1 µg/mL, detection of significant levels was achieved and considered optimal due to its economical representation (Figure 1C). For the pooled serum sample dilution, an OD value of 2.310—the highest—was obtained at a 1:2000 dilution level (Figure 1D). Significantly, the OPD solution produced higher OD values than TMB for the substrate sensitivity analysis (as shown by the two-tailed t-test analysis with *p*-value = 0.0001) (Figure 1E). These optimal parameters were employed for the remaining assay procedure.

### 3.2. Cut-Off Point Determination for the ELISA

Following the use of homologous and heterologous venoms for the specificity assay, tested inhibitor concentrations showed percent inhibition greater than 40% for all homologous venoms (specifically, 49.67 and 52.72% for *D. jamesoni* and *D. angusticeps*, respectively). However, heterologous venoms revealed a percent inhibition of less than 19% across all tested inhibitor concentrations (Table 1). Based on the deductions according to Sharma et al. [36], the specificity assay criteria for identifying the presence of an analyte of interest was set to 30% for both homologous and heterologous venom samples.

Furthermore, at various crude *D. polylepis* venom concentrations, the sensitivity of inhibition ELISA was determined, and serum samples from pre-immunized chickens (as negative controls) were analyzed. The limit of detection (LoD) was determined to be 0.01 µg/mL, using 0.973 and 0.202 recorded for the mean and standard deviation (of negative control), respectively (Table 2). The determined OD was 0.966, and thus fell in between the lowest assay concentration (0.02 µg/mL) and the NAC (No Antigen Control), given the 0.01 µg/mL as LoD. Hence, the ELISA was able to discriminate both positive and negative samples at a 0.01 µg/mL minimal concentration. Comparably, the assay could verify whether the specific toxins were present in a venom sample or not at the same cut-off. Lower optical density (OD) values indicate higher concentrations of the target analyte, which leads to increased competitive binding and a lower signal (OD), and vice versa, as is the case with inhibition ELISAs.

### 3.3. Assessment of Performance, Functionality and Overall Effectiveness of the Developed ELISA Prototype

#### 3.3.1. ELISA for Crude *D. polylepis* and Other Venoms Toxin Screening

**Figure 2:**
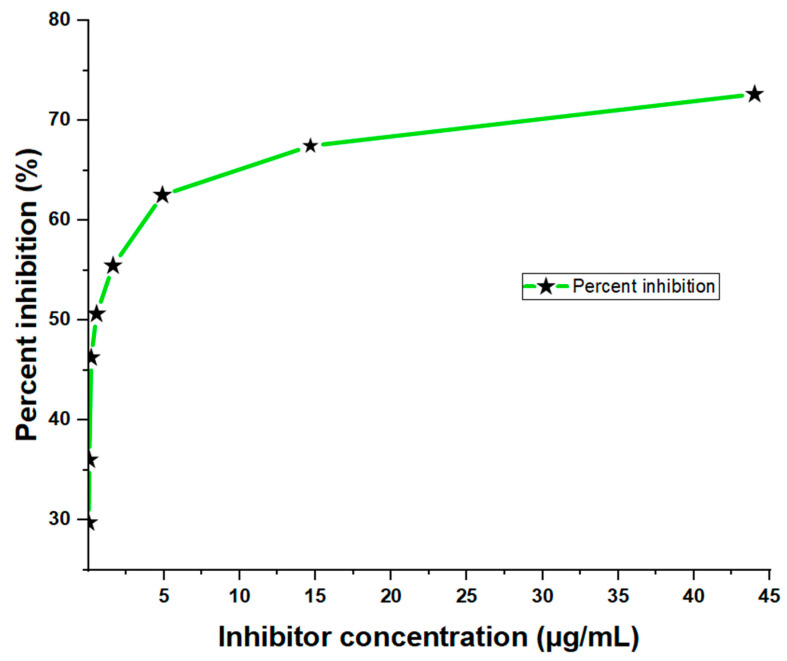
Dose-dependent ELISA inhibition curve showing percentages at sample-containing antigen levels of the inhibitor (crude *D. polylepis* venom).

From the three-fold dilution, the results showed significant inhibition of the sample antigen in a dose-dependent manner, with the highest inhibition (72.66%) observed at a concentration of 44.00 µg/mL and the lowest inhibition (29.78%) at 0.02 µg/mL (Figure 2). The corresponding absorbance values were 0.538 and 1.382, respectively. In contrast, the ‘No Antigen Control (NAC)’ sample showed a much higher absorbance value of 1.968 (Table 3), indicating a strong binding response in the absence of the antigen. The inhibition of toxins in the crude *D. polylepis* venom was observed across all concentrations, with the highest antigen concentrations showing the greatest reduction in OD values compared to the No Antigen Control (NAC). This suggests that the primary antibody bound more efficiently to the toxins at higher concentrations, resulting in increased percent inhibition.

From the toxin’s detection in venoms of *Dendroaspis* spp. and non-*Dendroaspis* spp., the inhibition ELISA assay revealed the presence of toxins in all assayed crude venoms and, at different tested concentrations, the percent inhibition due to sample antigens in various crude venoms was determined. Means of the sample antigen-induced inhibition were subjected to ANOVA and the result was statistically significant (*p* value = 0.0001), as shown in the table of ANOVA summary below (Table 4). Given the F-value, a higher variation between the samples means that relative to variation within the venom samples was observed. Additionally, the analysis showed R-squared = 0.6814, translating that sample antigen presence in crude venoms caused 68% of the variation in inhibition.

Tukey’s test results for multiple mean comparisons revealed significant differences in inhibition caused by toxins-containing samples between *Dendroaspis* spp. and non-*Dendroaspis* spp. Moreover, no significant difference was observed among all *Dendroaspis* species, as well as induced inhibition between *N. ashei* and *B. arietans* (Table 5).

**Figure 3:**
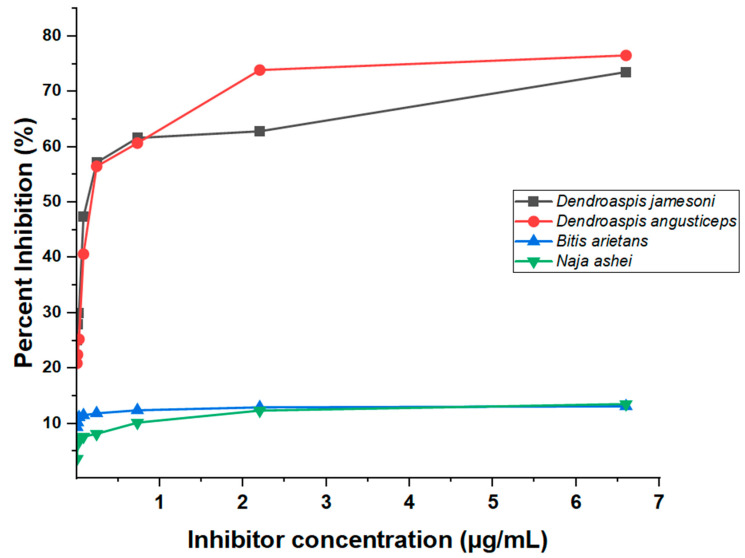
An ELISA inhibition curve showing percentages at sample-containing antigen levels of the inhibitors (crude *D. jamesoni, D. angusticeps, N. ashei* and *B. arietans* venoms at different concentrations).

This study further revealed low ODs for homologous species (*D. jamesoni* and *D. angusticeps*) at higher inhibitor concentrations resulting in high induction of percent inhibition, and OD increases with observed inhibitor-concentration reduction (Figure 3). The ODs of ‘No antigen control (NAC)’ for all venoms tested had the highest values recorded and was a sign of inhibition in contrast to wells with various concentrations of inhibitor. Since the venoms of *N. ashei* and *B. arietans* either contain little or none of the target antigens, the ODs observed for these two species indicate that there was either no inhibition or very little inhibition.

#### 3.3.2. Toxin Detection in *D. polylepis* Venom-Challenged Mice

**Figure 4:**
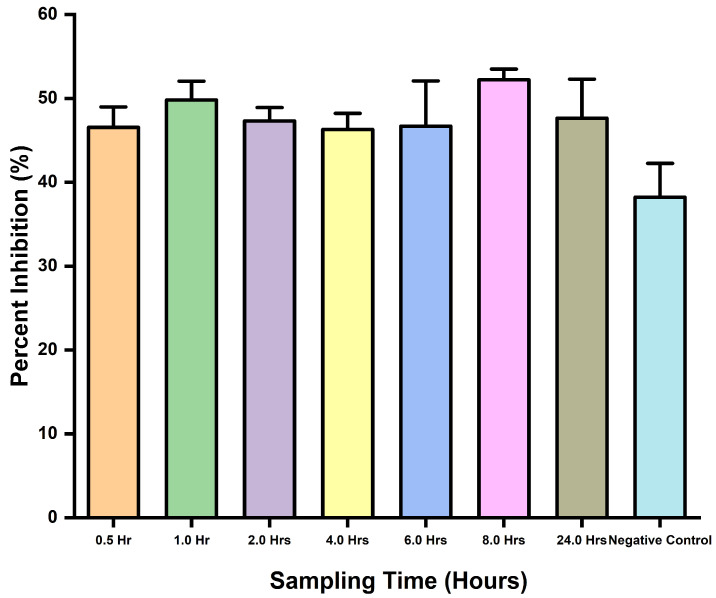
Toxins detection in venom-challenged mice, and percent inhibition comparisons for different time interval sampling.

This assay assessed the applicability of the inhibition ELISA potential to detect snakebite envenoming in mice using *D. polylepis* venom. It was clearly revealed by the ELISA’s ability to detect the toxins with observed inhibitions in wells containing the samples. There was no statistical difference (*p*-value = 0.3392) between the samples and negative control, and the same was observed for comparison tests through the one-way ANOVA. In addition, the assay was able to detect toxins with observed percent inhibition (Figure 4).

#### 3.3.3. Toxin Detection in Spiked Samples

**Figure 5:**
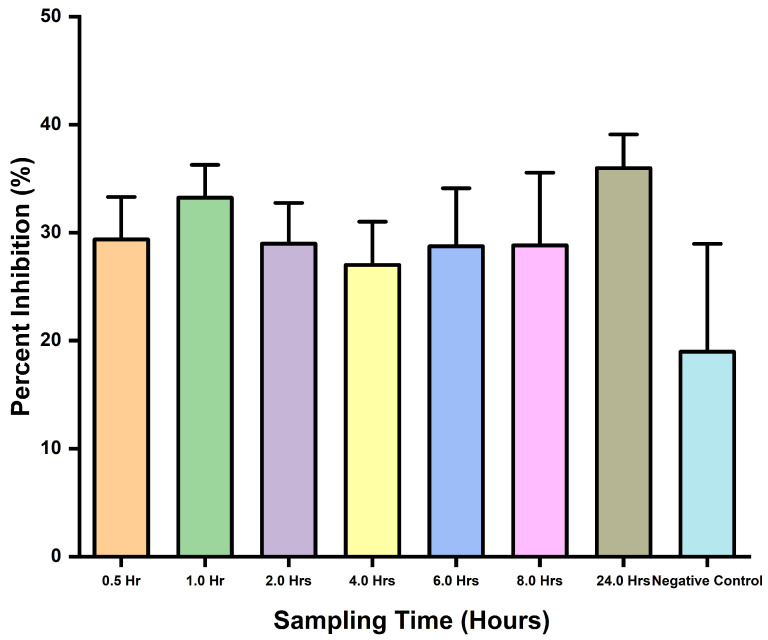
Different times sampling comparison for percent inhibition of spike samples collected.

Blood samples collected and spiked with *D. polylepis* venom at different times were assayed to assess the ELISA’s ability to detect venom toxins in blood. A control sample of previously collected serum (pre-immune) samples was assayed, along with blood samples. From the assay results, inhibition was observed but not for the negative control due to the absence of target antigen or antibodies. Like that of the previous assay, no significant difference (*p*-value = 0.0602) was observed from the ANOVA analysis with respect to the sampling times. However, the ELISA was able to detect the toxins in blood samples with some level of percent inhibition (Figure 5).

#### 3.3.4. Detection of Antibodies in Commercial Antivenom Products

**Figure 6:**
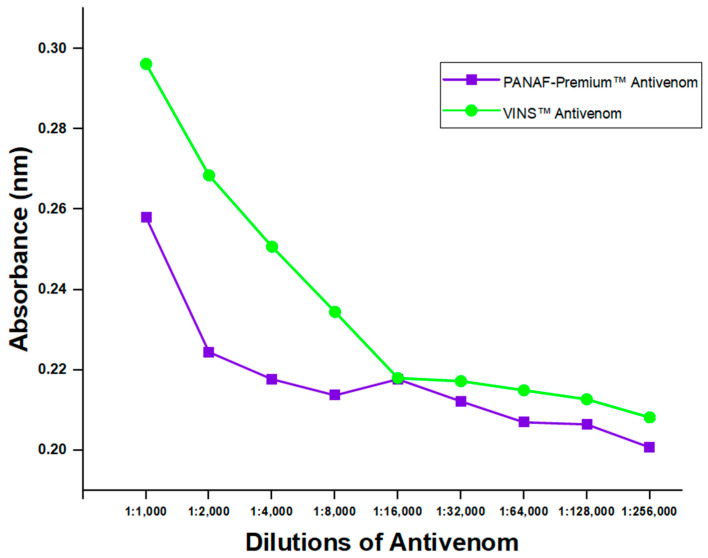
Anti-*D. polylepis* antibodies detection in two commercial antivenoms by the developed ELISA assay.

Two commercially available polyvalent antivenoms (PANAF-Premium^TM^ and VINS^TM^) were tested for the existence of toxins-specific antibodies using the developed indirect ELISA assay (thus, the assay’s ability to identify their presence in them). Despite the results showing higher detection for VINS^TM^, there were no varying detections observed beyond 1:16,000 dilution. It was revealed that antibodies in the antivenoms could be detected by the assay, even at the 1:256,000 dilution level (Figure 6). However, a comparison of the detection efficacy between PANAF-Premium^TM^ and VINS^TM^ showed significant differences at all dilution levels (*p*-value = 0.0001).

## 4. Discussion

This study details a sensitive ELISA for venom detection and quantification of one of Kenya’s snakes of medical importance. First and foremost, the development of an effective assay depends on a number of factors such as figuring out the best concentration for antigen coating, the ideal sensitive substrate and good primary/secondary antibody dilutions [36]. The optimal concentration for antigen coating found in this study is comparable to previous reports [13,37]. As the observations in research from Goka & Farthing [38] and Hosoda et al. [39] found that TMB substrate was very sensitive, this study had relatively higher absorbance for OPD substrate with a statistical difference from TMB, as also reported by Manson et al. [13]. Whereas the optimal dilution for secondary antibody (i.e., the detecting antibody) was consistent with Manson and associates, the observed optimal primary antibody dilution found in this study was contradictory.

Upon adopting the optimal conditions for further assays, the developed assay exhibited better analytical sensitivity with an observed lower limit of detection (LoD) when compared to prior published investigations. The increased sensitivity demonstrated is partly attributed to the use of easy-to-afford steps. More so, apart from the closely related *Dendroaspis* species, there was an observed minimum (less than 4%) of cross-reactivity between heterologous venoms. Despite this, the assay still enables the offending snake genus to be identified in these situations. Also, it is worth noting that since all *Dendroaspis* species induce identical clinical symptoms, there is little clinical or practical benefit to distinguishing between them [40,41]. Therefore, this assay will continue to be helpful in clinical decision making, even when antivenom for *Dendroaspis* becomes accessible. The ELISA results of the inhibition showed that, in comparison to NAC, the toxins found in the crude *D. polylepis* venom were inhibited at all tested concentrations with the reflection of reduced ODs, especially from high antigen-containing samples. In short, the high percent inhibition can be explained by the fact that there was more antigen in these wells, which led to increased binding of the primary antibody with the antigen.

Conversely, since the venoms of *N. ashei* and *B. arietans* either contain little or no of the target antigen, the ODs reported in this study for these two species indicate that there was no or very little inhibition. This may, however, be attributed to the established fact in reports that most of the toxins in these two species are highly cytotoxic, and do not seem to have most toxins that are neurotoxic, which distinguishes the *Dendroaspis* species [42,43]. Additionally, the observation by Casasola and associates that snake venoms belonging to the same genera exhibit similar biochemical, antigenic, and toxicological features supports the parallels in inhibition amongst the three *Dendroaspis* (*D. polylepsi*, *D. jamesoni* and *D. angusticeps*) snakes [44]. More so, the overall one-way ANOVA analysis showed R-squared (0.6814), translating that sample antigen presence in crude venoms caused 68% of the variation in inhibition. Also, the observed high F-value was an indication of a higher variation between the sample means relative to the variation within the venom samples when then amounted to the significant differences (*p*-value = 0.0001).

According to the observed variations in percentage inhibition when compared to the negative control, the expression of toxin proteins was detected by the ELISA in both blood samples of venom-challenged mice and ones spiked with crude *D. polylepis* venom. However, the spiked and venom-challenged blood samples when compared to the control were not statistically significant, but comparable to Manson et al. [13]. This could be attributed to the large similarity of the observed percent inhibition across the various sample collection times. In addition, the no significant difference seen between samples and control could be that the test run was just unable to detect the association or difference. Also, the ELISA was able to detect the existence of toxins-specific antibodies in two polyvalent antivenoms, which are for treating bites from varied snake species, including *D. polylepis*. These observations are quite significant because the assay was not only able to differentiate venoms but also help implicate offending snakes and help administer treatments targeted at specific venom toxins [45]. Reports on *D. polylepis* neutralization assays have indicated VINS product to be more effective among other polyvalent antivenoms [46,47] and are, however, consistent with our antibodies detection test for antivenoms. Comparison between the antivenoms displayed higher detections for VINS^TM^ with statistical significance, and could be speculated that the variances might be caused by differences in the protein concentrations of the batches of antivenom, among other factors [48]. The results found in our study, however, are limited to these particular batches; thus, caution should be used when interpreting them.

Since ELISA’s initial description in 1977 [19], ELISA-based snake venom detection has been applied in several laboratory, preclinical and clinical investigations [13,49]. ELISA has proven to be a useful tool when examining snake venom kinetics in blood, the extent of envenomation and the suitability of antigen-based serotherapy. Out of all the immunoassays, ELISA is thought to have greater practical usefulness and is still the preferred approach for identifying poisons, venoms and venom antibodies in bodily fluids [20,50,51]. The low LoD values found in this study are comparable to the reported LoD of a developed sensitive venom assay (ELISA) for African spitting cobra venom [13]. This then implies that when venom of *D. polylepis* (or *Dendroaspis* species) is injected in comparatively small amounts, it can be detected in preclinical serum samples due to the excellent analytical sensitivity of the ELISA method reported here. This can, however, be translated into clinical samples, thereby enabling the immunological diagnosis of envenoming from these species.

One major restriction of this developed ELISA is the cross-reactivity of the chicken-based IgY antibodies and venom between closely related species. The observed cross-reactivity is not out of the norm since similar in vivo toxic effects, venom characteristics and clinical envenoming signs have all been documented for homologous venom samples [34,49]. As a result, there was a significantly high amount of cross-reactivity between the venoms of the two *Dendroaspis* species and the *Dendroaspis polylepis* antibodies in this study. Nonetheless, this cross-reactivity was very minimal for the unrelated species, which suggests that the ELISA could readily distinguish between the viperid (*Bitis arietans*) and elapid (*N. ashei*) species among homologous ones. Since *D. polylepis* is responsible for the majority of *Dendroaspis* (mamba) cases in Kenya, the sameness of clinical effects/symptoms and the same therapeutic approach in cases by the three *Dendroaspis* species [40,52] should not have any significant implications for the application of developed ELISA in the clinical setting.

Despite the mamba being a medically important snake, especially in sub-Saharan Africa, and presenting many fatality issues, no documented report on the development of immunoassay for *Dendroaspis* species (mamba snakes’ venom) has been found yet. This study reports on the detection of the venom of *D. polylepis* from Kenya for the first time.

For epidemiological reasons, it is critical to identify the kind of offending snake’s venom present in patients’ blood. Also, antivenom dosing relies most importantly on the detection of snake venom in serum. A study from Sri Lanka indicates a syndromic technique or presentation of images is frequently used to identify the snake, and only approximately 25% of victims who are taken to hospitals have the specimens that caused the bite [24]. However, the validity of epidemiological investigations is limited, since none of these methods is precise enough. Because there is a chance of bites, guidelines for the treatment of envenomation from snakebite do not advise catching the offending snake and transporting it to the hospital. In numerous clinical investigations, the identification of snakebite cases has been hampered by the absence of venom confirmation [53,54]. Thus, enrolling envenomed patients in clinical investigations would be made easier with the use of venom-detecting ELISA, thereby increasing the correlation between clinical and therapeutic findings and snake identification. However, the lengthy and labor-intensive procedure for detecting snake venom antigens that are disclosed in this work must be carried out in a laboratory by a qualified technician, which presents a limitation. Therefore, in a clinical environment, the test proposed does not permit the prompt detection of venom. To identify the venom at the point of care, it would be feasible to design a quick venom detection kit, like an immunochromatography (lateral flow) test. Notwithstanding, the research offers evidence that it is possible to identify the venom of the most medically important snake in Kenya with precision. Also, this study focused on preclinical sera samples and encouraged the use of human sera for further studies. In the future, a rapid point-of-care venom detection kit in Kenya can benefit greatly from the knowledge provided in our research.

## 5. Conclusions

Conclusively, the assay-tested venoms of five snake species are categorized as “category 1”, meaning they pose the greatest risk to public health according to the Kenyan Ministry of Health and WHO.

The developed ELISA was capable of differentiating venoms from the species under study due to its analytical specificity and sensitivity, making it a potentially useful tool for the diagnosis and clinical treatment of envenomation caused by snakebite in Kenya. The findings suggest the possibility of developing venom detection kits with quick turnaround times based on liquid or lateral flow tests. Nonetheless, a thorough assessment of this assay’s usefulness in human envenomation is necessary. In order to further verify the assay’s sensitivity, more research on a variety of venoms might be required.

## Figures and Tables

**Table 1:** Detected percent inhibition by venoms of *D. jamesoni*, *D. angusticeps*, *B. arietans* and *N. ashei.*

Inhibitor Concentration (µg/mL)	*Dendroaspis jamesoni*		*Dendroaspis angusticeps*		*Bitis arietans*		*Naja ashei*	
	OD	% Inhibition	OD	% Inhibition	OD	% Inhibition	OD	% Inhibition
6.600	0.065	89.31	0.092	86.82	0.166	18.62	0.179	18.63
2.200	0.079	87.01	0.105	84.96	0.172	15.68	0.182	17.27
0.730	0.094	84.54	0.086	87.68	0.170	16.67	0.181	17.73
0.240	0.183	69.90	0.196	71.92	0.173	15.19	0.187	15.00
0.081	0.195	67.93	0.208	70.20	0.175	14.21	0.193	12.27
0.027	0.207	65.95	0.210	69.91	0.181	11.27	0.187	15.00
0.009	0.214	64.80	0.238	65.90	0.190	6.86	0.198	10.00
0.003	0.306	49.67	0.330	52.72	0.193	5.39	0.213	3.18
NAC	0.608		0.698		0.204		0.220	

**Table 2:** Percent inhibition for sensitivity assay determination.

Percent (%) Inhibition for ELSIA Sensitivity Determination				
*Dendroaspis polylepis* Venom			Negative Controls (n = 16)	
Concentration (µg/mL)	Replicate 1	Replicate 2		
44.00	76.00	80.61	50.06	48.28
14.67	68.85	72.65	47.08	46.95
4.89	67.06	65.51	44.97	44.70
1.63	65.87	59.79	42.58	44.10
0.54	64.88	57.96	34.77	37.02
0.18	50.20	54.49	32.18	34.44
0.06	42.06	50.82	20.46	21.39
0.02	39.08	45.92	12.58	11.26

**Table 3:** Antigen-mediated inhibition of toxins from crude *D. polylepis* venom.

Inhibitor Concentration (µg/mL)	OD (492 nm)	Percent Inhibition (%)
44.00	0.538	72.66
14.67	0.640	67.48
4.89	0.737	62.55
1.63	0.876	55.49
0.54	0.971	50.66
0.18	1.057	46.29
0.06	1.258	36.07
0.02	1.382	29.78
NAC	1.968	-

**Table 4:** Overall one-way ANOVA results ^m^.

	DF	Sum of Squares	Mean Square
Model	4	1.86281	0.4657
Error	35	0.8709	0.02488
Total	39	2.7337	-
F value		18.7159	
*p* value		<0.0001	
*p* value summary		****	
Significant difference among means (*p* < 0.05)?		Yes	
R-squared		0.6814	

^m^ The overall ANOVA table depicting statistical significance exists or not for the inhibition caused by *Dendroaspis* spp. and non-*Dendroaspis* spp. venoms containing samples. The goal of this experiment was to assess the assay’s capacity for specific detection of homologous (*D. jamesoni*, *D. angusticeps* and *D. polylepis*) venom samples while maintaining a high degree of discrimination against heterologous ones (*N. ashei* and *B. arietans*). DF means the degrees of freedom in the source. **** indicates significance.

**Table 5:** Tukey’s multiple comparison test results ^n.^

Tukey’s Multiple Comparison Test	Adjusted *p* Value
*D. jamesoni* vs. *D. angusticeps*	0.99829
*D. jamesoni* vs. *B. arietans*	0.03341
*D. angusticeps* vs. *B. arietans*	0.02264
*N. ashei* vs. *D. jamesoni*	0.00206
*N. ashei* vs. *D. angusticeps*	0.00132
*B. arietans* vs. *N. ashei*	0.67821
*B. arietans* vs. *D. polylepis*	<0.0001
*D. polylepis* vs. *D. jamesoni*	0.34015
*D. polylepis* vs. *D. angusticeps*	0.09557
*D. polylepis* vs. *N. ashei*	<0.0001

An adjusted *p* value < 0.05 is significantly different. ^n^ the venom samples of homologous species and heterologous species were compared against one another and were carried out using Tukey’s multiple comparison test from ANOVA to examine the sample-induced inhibition differences.

## Data Availability

This report contains all of the data that were used, and the corresponding author can be contacted for additional information.
